# Enhancement of cellular glucose uptake by reactive species: a promising approach for diabetes therapy

**DOI:** 10.1039/c7ra13389h

**Published:** 2018-03-08

**Authors:** Naresh Kumar, Priyanka Shaw, Jamoliddin Razzokov, Maksudbek Yusupov, Pankaj Attri, Han Sup Uhm, Eun Ha Choi, Annemie Bogaerts

**Affiliations:** Department of Chemistry, University of Antwerp Universiteitsplein 1, B-2610 Antwerp Belgium nash.bms@gmail.com chem.pankaj@gmail.com annemie.bogaerts@uantwerpen.be +32-(0)3-265-23-43 +32-(0)3-265-23-81; Plasma Bioscience Research Center/Department of Electrical and Biological Physics, Kwangwoon University 20 Kwangwon-Ro, Nowon-Gu Seoul 139-701 Korea

## Abstract

It is generally known that antidiabetic activity is associated with an increased level of glucose uptake in adipocytes and skeletal muscle cells. However, the role of exogenous reactive oxygen and nitrogen species (RONS) in muscle development and more importantly in glucose uptake is largely unknown. We investigate the effect of RONS generated by cold atmospheric plasma (CAP) in glucose uptake. We show that the glucose uptake is significantly enhanced in differentiated L6 skeletal muscle cells after CAP treatment. We also observe a significant increase of the intracellular Ca^++^ and ROS level, without causing toxicity. One of the possible reasons for an elevated level of glucose uptake as well as intracellular ROS and Ca^++^ ions is probably the increased oxidative stress leading to glucose transport.

## Abstract 2

Influenence of biocompatible microsecond dielectric barrier discharge (μs-DBD) plasma in glucose uptake and cell differentiation.

## Graphical abstract

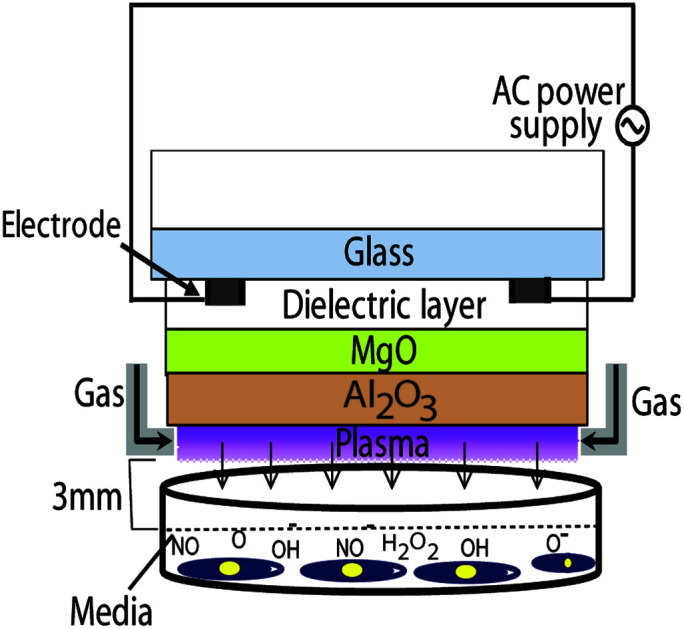

## 1. Introduction

Approximately 75% of insulin-stimulated glucose uptake in our body is performed by skeletal muscles. Impairment of glucose uptake by skeletal muscle tissue causes a high level of sugar presence in the blood. This is because either the insulin production is inadequate or the body cells do not respond properly to the insulin, or both,^1–3^ which eventually leads to a diabetic disease. Currently available drugs for treatment of diabetics are often ineffective and there is a need for an alternative remedy that should not depend on insulin-based glucose uptake, due to insulin resistance in muscles.

There are few experimental investigations in the literature where insulin-independent glucose uptake was studied based on muscle contraction and electrical stimulation of muscles, although the underlying mechanisms in these studies are poorly understood.^4–6^

Thus, the main objective of the current study is to find out the molecular level mechanisms of glucose uptake that regulate the glucose homeostasis. For this purpose, we employ a new method based on cold atmospheric plasma (CAP). CAP sources are known to generate a cocktail of positive and negative ions, free radicals, neutrals, *etc.*^7–11^ CAPs are nowadays used in different fields, such as disinfection of both living tissue and non-living surfaces (*e.g.*, medical tools and diagnostic devices),^12^ treatment of chronic wounds^13^ and even killing or apoptosis of cancer cells.^14^ Recent reports suggest that CAP with lower doses stimulates macrophages to release various cytokine and tumour necrosis factor-alpha, which contribute to the selective killing of a variety of cancer cells.^15,16^ It also promotes stem cell differentiation,^17,18^ enhances cell transfection^19^ and possibly increases wound healing.^20^ Reactive oxygen and nitrogen species (RONS) generated by CAP selectively initiate and amplify ROS signalling to enhance osteoblast proliferation and differentiation.^7^

Recently we investigated that NO species generated by a microwave plasma torch increase myogenesis and significantly elevate the expression of myogenic precursor genes, such as myoD, MHC, and myogenin, which indicates the formation of new muscle tissue.^21^ However, the application of CAP in diabetes mellitus and skeletal muscle cell differentiation investigation are in primarily stage, although it is most likely that CAP might play a role in regulating glucose uptake and myoblast differentiation.

Hence, in order to understand the glucose uptake processes by muscle cells as well as cell differentiation, we apply biocompatible microsecond dielectric barrier discharge (μs-DBD) plasma.

## 2. Experimental details

### 2.1. Materials

Dulbecco's modified eagle medium (DMEM) and fetal bovine serum (FBS) are purchased from Life Technologies. Skeletal muscle L6 cell lines procured from KCLB (Korean Cell Line Bank, South Korea), are used in the experiments. Exogenous H_2_O_2_ and NO are measured using an Amplex H_2_O_2_ assay kit (Invitrogen, USA) and a NO detection assay kit (Biovision, USA), respectively. H_2_DCFDA (2′,7′-dichlorodihydrofluorescein diacetate; Invitrogen) is used for the detection of intracellular ROS. OH and H_2_O_2_ scavengers, *i.e.*, 6-hydroxy-2,5,7,8-tetramethylchroman-2-carboxylic acid (trolox), are purchased from Sigma Aldrich. Anti-alpha tubulin antibody, FITC conjugated tubulin and rhodamine-phalloidin, nuclear stain DAPI, are purchased from Abcam USA. A fluorescent probe of glucose, *i.e.*, 2-[*N*-(7-nitrobenz-2-oxa-1,3-diazol-4-yl) amino]-2-deoxy-d-glucose (2NBDG), is used to study the glucose uptake, which is purchased from Invitrogen, USA.

### 2.2. Rat skeletal muscle cells (L6) culture

L6 myoblasts are used to study the cell proliferation and differentiation in a 6-well plate after treatment with the μs-DBD. The cells are cultured in DMEM and supplemented with 10% FBS and 1% penicillin–streptomycin (p/s) under standard culture conditions (37 °C, 5% CO_2_). The cells are seeded onto the 6-well plate at a density of 5 × 10^4^ cell per ml and cultured under growth media for 24 h. To induce myotube formation, the media are then replaced with differentiation media (DMEM supplemented with 2% horse serum and 1% p/s). The cells are exposed to μs-DBD for 30, 60, 90, 120 and 180 s. Untreated media are included as a 0 min treatment in each experiment. After exposure, on day 2, 4, and 8 the treated L6 cells are used to check the viability through MTT (3-[4,5-dimethylthiazol-2yl]-2,5-diphenyltetrazolium bromide) assay.^21^

### 2.3. Microsecond dielectric barrier discharge (μs-DBD) plasma device properties and cell treatment

**Fig. 1:**
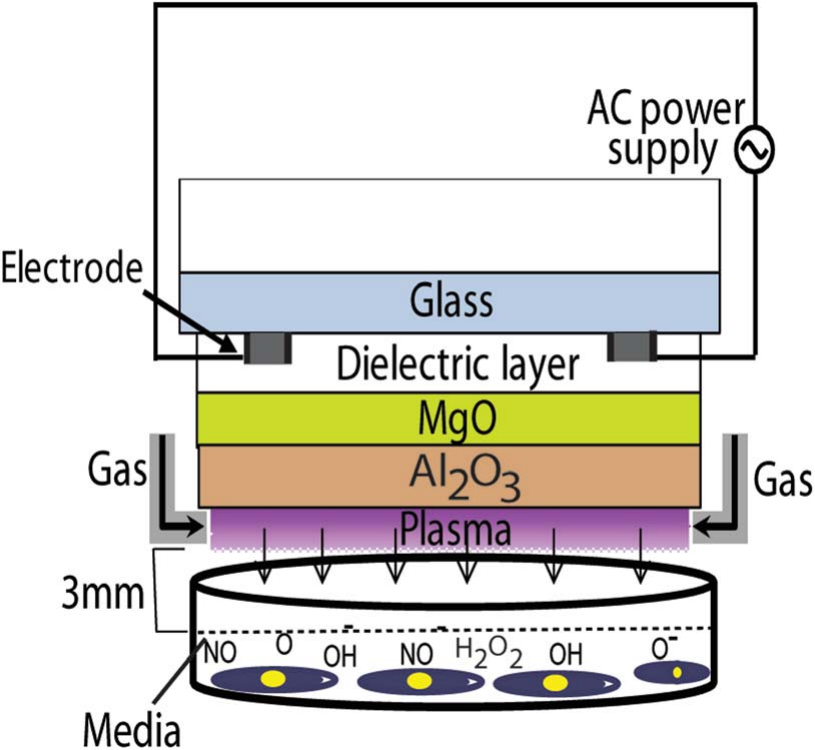
Schematic diagram of the μs-DBD device. Nitrogen is used as a feeding gas with a flow rate of 1 SLM.

In this study we use a μs-DBD device, previously reported and shown in Fig. 1.^12,15^

It consists of two electrodes, a dielectric layer (silicon dioxide (SiO_2_)) and hydration prevention layers made of aluminium oxide (Al_2_O_3_) and magnesium oxide (MgO). The electrodes have a thickness of 5 μm and a width of 200 μm, and they are separated by a 200 μm gap. Diameter of the plasma discharge area is about 35 mm. To generate plasma, nitrogen gas was injected into the device with a flow rate of 1 standard litre per minute (SLM). A DC–AC inverter system is used for power supply with about 30 V of primary input voltage. Due to transformer of DC–AC inverter, the frequency of AC power supply is about 30 kHz (see Fig. 1). After confluence of 70%, the L6 skeletal muscle cells are treated with μs-DBD plasma for 30, 60, 90, 120 and 180 s. A constant gap of 3 mm between the plasma source and the upper surface of the cell culture media is maintained in all experiments. The working temperature of the plasma source is in the range of 20–26 °C. More detailed information about the design and operation of the μs-DBD plasma device is given in ref. 15.

### 2.4. Physical and chemical change in the culture media

After exposure of DMEM in a 6-well plate (2 ml per well) to the μs-DBD plasma, the pH and temperature of the media are measured using a pH meter (Eutech Instruments, Singapore) and Infrared (IR) camera (Fluke Ti100 Series Thermal Imaging Cameras, UK), respectively. Simultaneously, RONS levels in DMEM media were analyzed after treated with μs-DBD plasma for 30, 60, 90, 120 and 180 s. The amounts of OH and H_2_O_2_ were measured as per previous described methods,^14^ while NO, & NO_2_^−^ was measured using the chemical kit.

### 2.5. Measurement of protein content

The total protein content is measured at the designated time points (*i.e.*, on day 2, 4 and 8), after exposing the cells (in media) to the μs-DBD. All of the cell protein extracts from the treated/untreated cells are lysed in a radioimmunoprecipitation assay (RIPA) buffer (Cell Signaling Technology, USA) and the extracted proteins and protein concentration are measured using the protein assay kit (Biorad, USA).

### 2.6. Immunofluorescence staining

After incubation for 8 days, the treated muscle cells are used to check the cell morphology. The cells on the cover glass are immunofluorescence stained for α-tubulin and actin, which are visualized using a fluorescence microscope (TE-2000, Nikon Corp., Tokyo, Japan). The seeded cells are fixed in 4% of paraformaldehyde in phosphate buffered saline (PBS) and then permeabilized in cytoskeleton buffer (pH 6.8, 50 mM NaCl, 150 mM sucrose, 3 mM MgCl_2_, 50 mM Trizma-base, 0.5% Triton X-100). After permeabilization, non-specific binding sites are blocked by incubation with 5% FBS in PBS, and then sequentially incubated with FITC-conjugated α-tubulin (1 : 50). Afterwards, the cell nuclei and actin are stained with DAPI (1 : 5000) and rhodamine-phalloidin (1 : 200).

### 2.7. 2NBDG glucose uptake

Glucose uptake studies are carried out by using a fluorescent probe of glucose (2NBDG).^22^ We performed two sets of experiments in a 6-well plate. One set is used for quantification of the 2NBDG uptake. In the other set, the cells on the cover glass are used for qualitative fluorescence imaging. The cells are treated with the μs-DBD for 90 s with and without the presence of 100 μM insulin and the glucose uptake is measured on day 2, 4 and 8, using 80 μM 2NBDG fluorescent glucose analogue. After 60 min incubation at 37 °C with 2NBDG, the cells are washed three times with PBS and then quantified at 485/535 (ex./em.) nm using a microplate reader. The mean fluorescence intensity is calculated between the plasma-treated and control (*i.e.*, untreated) cells. For fluorescence, the image cells are visualized using a fluorescence microscope (TE-2000, Nikon Corp., Tokyo, Japan).

### 2.8. Measurement of intracellular calcium levels

To study the cytoplasmic calcium (Ca^++^) level, L6 cells in DMEM are treated with the μs-DBD for 90 s with and without the presence of membrane-permeant Ca^++^ chelator 1,2-bis-(2-aminophenoxy)ethane-*N*,*N*,*N*′,*N*′-tetraacetic acid tetra(acetoxymethyl) ester (BAPTA) on day 2, 4 and 8 in a 6-well plate. The Ca^++^ concentration in the supernatant material is estimated by the Calcium Detection Kit (abcam, USA). We followed the standard protocol according to the datasheet. For intracellular calcium images, we separately seed the cells on the cover glass in DMEM and treat the cells with the μs-DBD for 90 s and 90 s + BAPTA/AM. On day 8 of incubation the cells are rinsed with assay buffer (130 mM NaCl, 10 mM glucose, 5 mM KCl, 2 mM CaCl_2_, 1.2 mM MgCl_2_, and 10 mM HEPES, pH 7.4) and loaded with 5 mM Fura-2 AM (Invitrogen) for 30 min at room temperature. The cells are visualized using a fluorescence microscope.^23,24^

### 2.9. Quantitative analysis of intracellular ROS content

To study the total ROS content inside the cells, we use the H_2_DCFDA probe. The attached L6 cells in DMEM are treated with the μs-DBD for 90 s and the intracellular ROS content is measured on day 2, 4 and 8 with and without the presence of 1 μM trolox (ROS scavenger), using 10 mM of H_2_DCFDA to each sample, and kept at 30 °C incubation for 1 h. Subsequently, the cells are washed twice with PBS and the intracellular ROS content is quantified at 495/515 (ex./em.) nm using a microplate reader. The mean fluorescence intensity is calculated between the plasma-treated and control (*i.e.*, untreated) cells.

### 2.10. Statistical analysis

All data values were shown as mean ± standard deviation (SD). Comparison of all other results was performed by one-way analysis of variance (ANOVA) with Tukey's comparison analysis. The data was considered significantly different when *p < 0.05, **p < 0.01, ***p < 0.001. Prism (Graphpad Software Inc.) and Excel Software (Microsoft Inc.) was used to compare the groups.

## 3. Results

### 3.1. Changes in physical and chemical properties of culture media after μs-DBD treatment

**Fig. 2:**
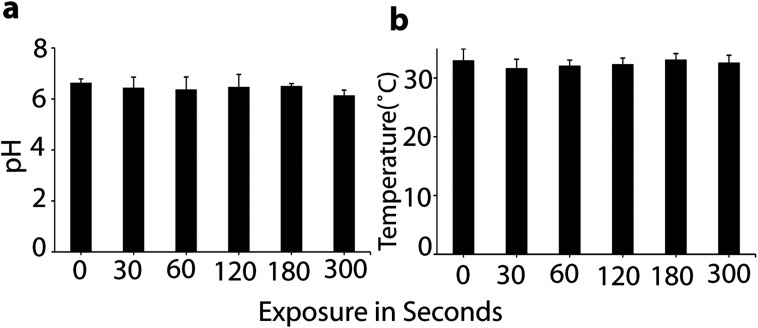
Measurements of pH (a) and temperature (b) after plasma treatment of the cell media. All values are expressed as ±SD in triplicates.

To determine the changes in the properties of the culture media after exposure to plasma, we applied the μs-DBD for 0, 30, 60, 120, 180 and 300 s. Fig. 2 shows no significant changes in pH and temperature.

**Fig. 3:**
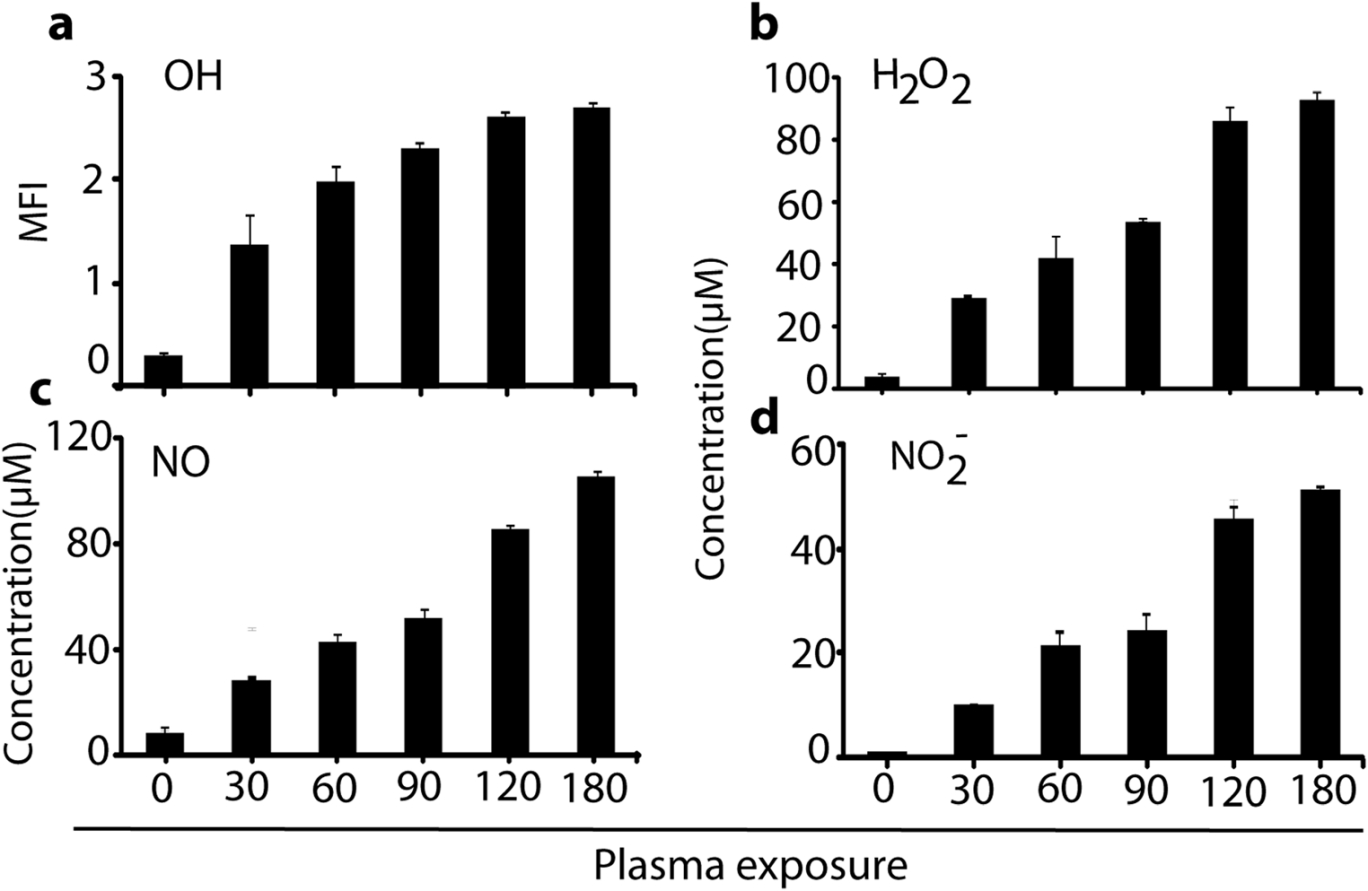
Measurements of the RONS content after plasma treatment of the cell media. Determination of (a) OH, (b) H_2_O_2_, (c) NO and (d) NO_2_^−^ concentrations in 2 ml media after μs-DBD plasma exposure at different times. All values are expressed as ±SD in triplicates.

As is obvious, the temperature of the media was maintained at around 32 °C during μs-DBD exposure, but the pH of the media slightly decreased after 300 s. These data indicate that up to 180 s, the pH and temperature have no adverse effect on the cell viability. Further, in order to measure the concentration of extracellular RONS in the media, we applied the same different time points (*i.e.*, 0, 30, 60, 120 and 180 s) which were used for the determination of pH and temperature in the culture media. Fig. 3 shows the concentrations of the RONS in the media treated with plasma up to 180 s.

As is clear, a high amount of OH, H_2_O_2_, NO, and NO_2_^−^ species inside the solution is observed in comparison to untreated (0 s) media. Indeed, 180 s of plasma treatment leads to a production of H_2_O_2_, NO and NO_2_^−^ of about 90, 100 and 55 μM, respectively. However, at a lower plasma dosage (*e.g.*, 60 s), the RONS are generated in moderate quantity, which might influence the cell differentiation as well as the glucose uptake. To examine this, we analysed the plasma effect on the cell viability, proliferation and toxicity, to determine at which exposure time the plasma triggers the elevation of cell differentiation (see next section).

### 3.2. μs-DBD plasma effects on cell viability and dose optimization

**Fig. 4:**
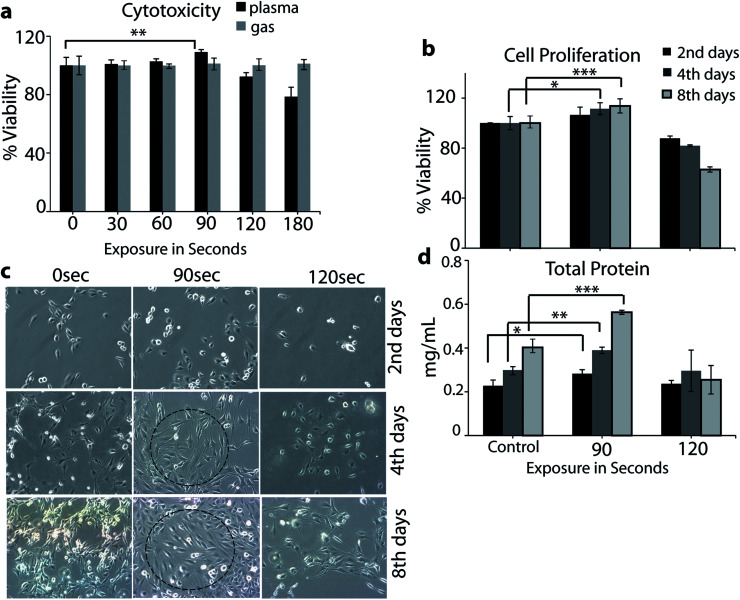
Analysis of L6 cell cytotoxicity (a), proliferation (b), differentiation (c) and total protein content (d). The cell differentiation analysis was performed using light microscopy, whereas the measurement of the total protein content was carried out on day 2, 4 and 8 after exposure to μs-DBD. Comparison between all groups showed significant differences by one-way ANOVA (* denotes *P* < 0.05, ** denotes *P* < 0.01 and *** denotes *P* < 0.001).

To find the plasma treatment time and dose at sub-lethal level, the cell viability was studied at different plasma doses. For determination of the cell viability, we performed a MTT assay. The L6 cells showed a significant decrease in viability after treatment with plasma for 120 and 180 s (*i.e.*, above 90 s) during 24 h of incubation time (see Fig. 4a). However, in the case of only air flow (*i.e.*, no plasma), we did not observe any influence on the cell viability. The MTT assay data showed that the plasma has no inhibitory effect on the growth of the L6 cells up to 90 s. Thus, in the rest of our experiments we used 90 and 120 s of exposure time to check the cell proliferation. It is clear from Fig. 4b that 90 s plasma exposure to the L6 cells showed significant proliferation on day 2, 4 and 8, whereas in the case of 120 s, the toxicity of the plasma exposure was noted in the cells (see Fig. 4b). The light microscope images given in Fig. 4c show clear cellular differentiation in the case of 90 s plasma exposure on day 2, 4 and 8 (see dashed circles).

This indicates that the plasma treatment for 90 s significantly affects the cellular differentiation on every consecutive day, while plasma exposure for 120 s represents toxicity (see Fig. 4c). The total protein content of the cells after treatment with the μs-DBD for 90 s was greater than that of the untreated cells and the cells treated with 120 s (see Fig. 4d). Under the exposure of 90 s, confluence of the myoblast cell layers was observed after 2 days of culturing, which continued to increase after 4 and 8 days. Hence, the total protein content indicates that the 90 s exposure of the cells to the μs-DBD plasma induces cell proliferation.

### 3.3. Influence of the μs-DBD plasma on cellular differentiation

**Fig. 5:**
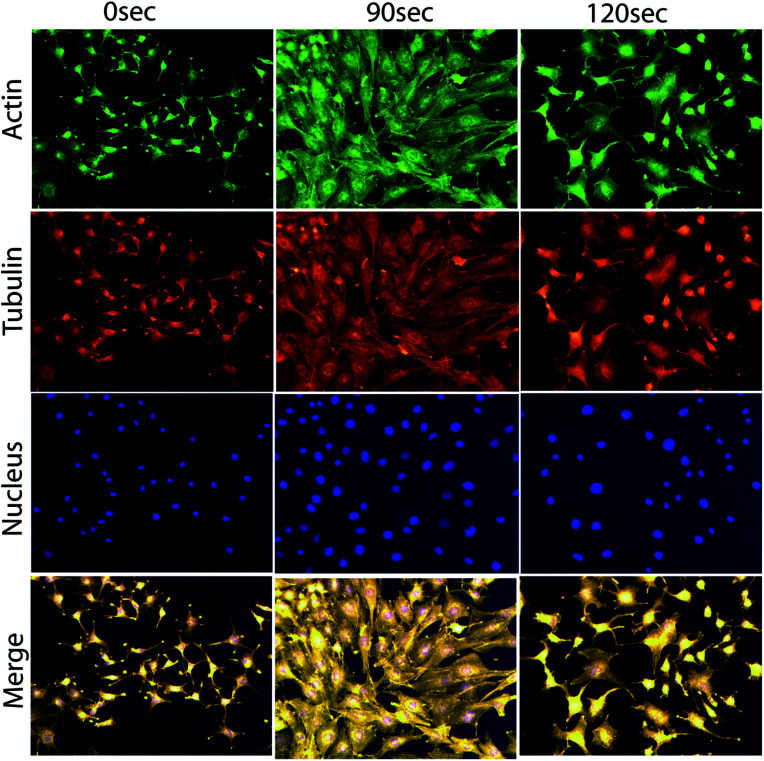
Cellular differentiation analysis through actin, tubulin and nuclear immunofluorescence images of L6 cells on day 8 after exposure to the μs-DBD.

For a more clear observation of the myoblast differentiation after plasma exposure, we performed immunostaining of actin and α-tubulin filaments on day 8 of incubation. The average area of the individual myotube was stained with monoclonal antibodies of α-tubulin conjugated with fluorescein isothiocyanate (FITC), and the actin filament was stained with rhodamine phalloidin. During the extended cell culture, the L6 myoblast showed a highly organized structure after differentiation into myotubes (Fig. 5), In particular, the majority of the cells cultured on day 8 of incubation showed that the differentiated myotubes were longer and wider in the case of 90 s compared to those of 0 and 120 s.

### 3.4. Plasma stimulated glucose uptake, intracellular Ca^++^ and ROS estimation

**Fig. 6:**
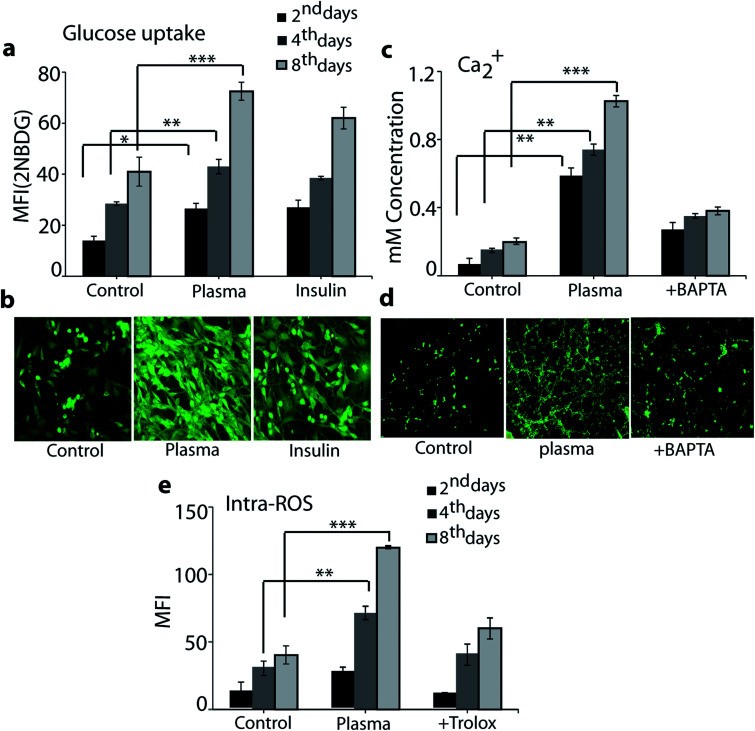
Glucose uptake (a) and (b), cytosolic Ca^++^ (c) and (d) and intracellular ROS (e) analysis after treatment with the μs-DBD for 90 s as well as with insulin (see (a) and (b)), chelator BAPTA (see (c) and (d)) and trolox (see (e)), measured on day 2, 4 and 8. Fluorescence images of 2NBDG uptake (b) and Ca^++^ (d) were taken on day 8. Comparison between all groups showed significant differences by one-way ANOVA (* denotes *P* < 0.05, ** denotes *P* < 0.01 and *** denotes *P* < 0.001).

To evaluate the efficacy of plasma in an enhancement of the glucose uptake in differentiated myoblasts, we treated the cells with plasma for 90 s and analysed the results on day 2, 4 and 8. Fig. 6a and b shows that the plasma treatment resulted in a significant increase in glucose uptake compared to basal levels: the intracellular level of 2NBDG (*i.e.*, fluorescent glucose indicator) increased in differentiated myotube over an incubation time of 2, 4 and 8 days, respectively (see Fig. 6a).

However, 100 nM of the insulin, which is used as a standard drug, also showed enhanced level of glucose uptake in comparison to control (*i.e.*, without plasma exposure), see Fig. 6a and b. The fluorescent image depicted in Fig. 6b shows that 8 days of incubation has a higher 2NBDG uptake for plasma treatment in comparison to the control and the case of insulin usage. As mentioned in the Introduction, plasma sources (including μs-DBD) generate a variety of RONS and these extracellular reactive species are able to penetrate directly or indirectly through the cell membrane, which eventually causes a change in membrane polarity and triggers oxidative stress.^25^ Hence, to observe the influence of plasma on the cell polarity change as well as on the total intracellular ROS level, we analysed the intracellular Ca^++^ and intracellular ROS content on day 2, 4 and 8 in differentiated myotube. Fig. 6c and d shows the intracellular level of Ca^++^ ions. As is clear, in the plasma treatment case the Ca^++^ level increased in differentiated myotube over an incubation time of 2, 4 and 8 days, compared to the control case. On the other hand, plasma exposure in the presence of 50 μM calcium inhibitor BAPTA showed no significant levels of Ca^++^, which proves that plasma exposure enhances the Ca^++^ levels. The fluorescent image illustrated in Fig. 6d also shows that 8 days of incubation resulted in a higher level of Ca^++^ fluorescence in the plasma treatment case compared to the control and the presence of calcium inhibitor BAPTA. We used the H_2_DCFDA diacetate fluorescent probe to detect the total intracellular ROS level. As is clear from Fig. 6e, 90 s of plasma treatment significantly increased the ROS level in comparison to the control and the presence of 50 μM trolox (*i.e.*, ROS scavenger) on day 2, 4 and 8. These results indicate that the increase of calcium and intracellular ROS level might play a role in stimulation of sugar uptake in the L6 cells as well as in their differentiation.

## 4. Discussion

The main objective of this study was to examine *in vitro* the plasma effects on glucose uptake through the biocompatible μs-DBD plasma, aiming at healing diabetic diseases. In addition to the study of glucose uptake, we investigated the differentiation of myoblastic rat L6 skeletal muscle cells. The L6 cell line can reproduce myogenic differentiation in the presence of a growth factor in a culture medium and has been the most widely used model to investigate myogenic differentiation and physical stress stimulated glucose uptake.^26,27^ Initially, we determined the specific μs-DBD plasma conditions, such as pH, temperature, extracellular RONS content and sub-lethal dose of plasma that is required to maintain the cell viability in response to a certain treatment time of plasma. It was found that 90 s exposure of μs-DBD plasma increases the cell proliferation, differentiation as well as total protein content (see Fig. 4). Additionally, the μs-DBD treatment also leads to an increase of the intracellular ROS and Ca^++^ content, as shown in Fig. 6. Previous papers showed that an increase of intracellular ROS content can activate the ion flux through the cell membrane, which influences the intracellular Ca^++^ concentration.^28^ An increase of Ca^++^ concentration activates the signal transduction pathway that helps to increase the glucose uptake.^29,30^ Furthermore, other research studies also revealed that oxidative stress caused by various factors, such as physical effects, electrical stimulation, *etc.*, help to increase the glucose uptake through an oxidative pathway.^4–6,31,32^ Hence, an increase of the level of intracellular reactive species as well as Ca^++^ ions helps to increase the glucose uptake by skeletal muscle cells. This is in line with our experimental results that an increase of intracellular ROS and Ca^++^ concentration, after plasma treatment, results in an increase of the glucose uptake. Thus, our experimental results might follow the above mentioned mechanism for glucose uptake. Note that there might be other pathways that also help to increase the glucose uptake after plasma treatment. Therefore, further study is required to explain the exact mechanism of glucose uptake induced by plasma treatment.

## 5. Conclusion

Our findings are highly important, since this investigation provides the first evidence of the antidiabetic potential of CAP, as it significantly stimulates glucose uptake and cellular differentiation in L6 skeletal muscle cells. Thus, CAP sources could further be developed for potential usage in antidiabetic therapy. This study is not solely important for diabetes, but might also be essential in cancer treatment, as recent investigations indicate that diabetic patients have 20–25% more risk of developing cancer compared to individuals without diabetes.^33,34^

## Conflicts of interest

The authors declare no conflict of interest.

## Supplementary Material
